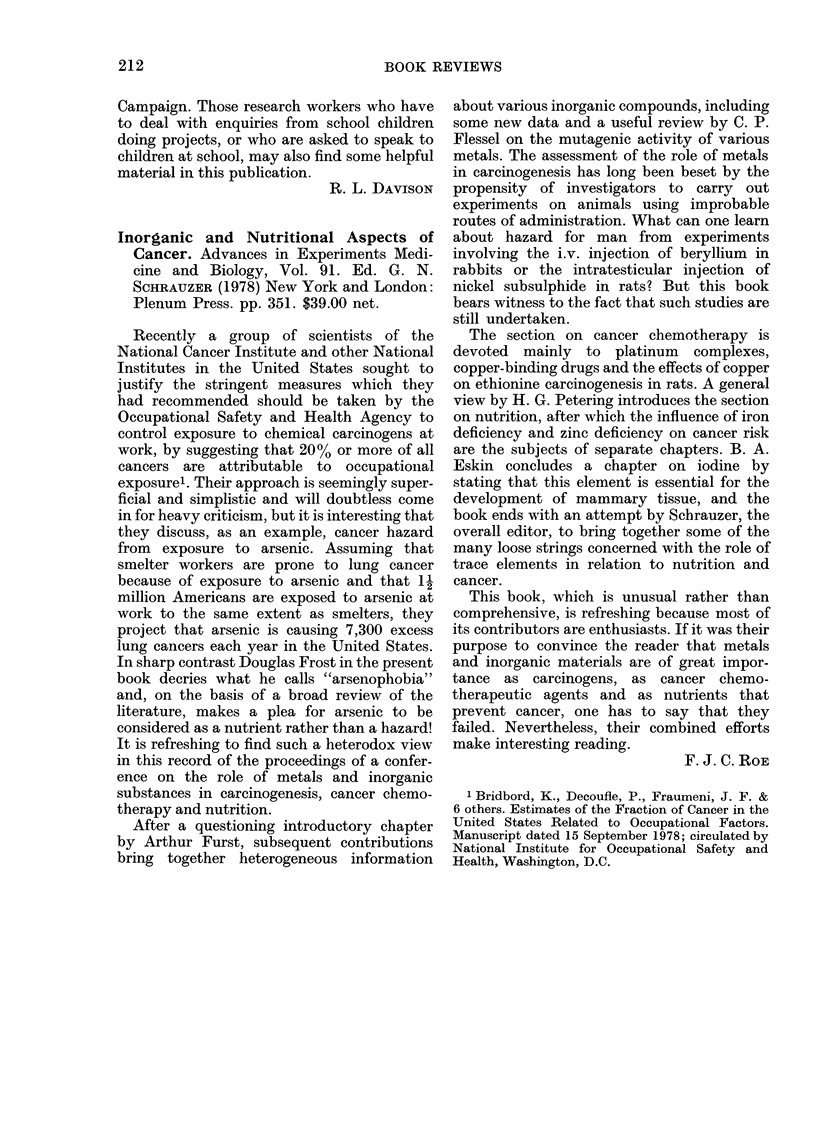# Inorganic and Nutritional Aspects of Cancer

**Published:** 1979-02

**Authors:** F. J. C. Roe


**Inorganic and Nutritional Aspects of Cancer.** Advances in Experiments Medicine and Biology, Vol. 91. Ed. G. N. SCHRAUZER (1978) New York and London: Plenum Press. pp. 351. $39.00 net.

Recently a group of scientists of the National Cancer Institute and other National Institutes in the United States sought to justify the stringent measures which they had recommended should be taken by the Occupational Safety and Health Agency to control exposure to chemical carcinogens at work, by suggesting that 20% or more of all cancers are attributable to occupational exposure[1]. Their approach is seemingly superficial and simplistic and will doubtless come in for heavy criticism, but it is interesting that they discuss, as an example, cancer hazard from exposure to arsenic. Assuming that smelter workers are prone to lung cancer because of exposure to arsenic and that $1\frac{1}{2}$ million Americans are exposed to arsenic at work to the same extent as smelters, they project that arsenic is causing 7,300 excess lung cancers each year in the United States. In sharp contrast Douglas Frost in the present book decries what he calls "arsenophobia" and, on the basis of a broad review of the literature, makes a plea for arsenic to be considered as a nutrient rather than a hazard! It is refreshing to find such a heterodox view in this record of the proceedings of a conference on the role of metals and inorganic substances in carcinogenesis, cancer chemotherapy and nutrition.

After a questioning introductory chapter by Arthur Furst, subsequent contributions bring together heterogeneous information about various inorganic compounds, including some new data and a useful review by C. P. Flessel on the mutagenic activity of various metals. The assessment of the role of metals in carcinogenesis has long been beset by the propensity of investigators to carry out experiments on animals using improbable routes of administration. What can one learn about hazard for man from experiments involving the i.v. injection of beryllium in rabbits or the intratesticular injection of nickel subsulphide in rats? But this book bears witness to the fact that such studies are still undertaken.

The section on cancer chemotherapy is devoted mainly to platinum complexes, copper-binding drugs and the effects of copper on ethionine carcinogenesis in rats. A general view by H. G. Petering introduces the section on nutrition, after which the influence of iron deficiency and zinc deficiency on cancer risk are the subjects of separate chapters. B. A. Eskin concludes a chapter on iodine by stating that this element is essential for the development of mammary tissue, and the book ends with an attempt by Schrauzer, the overall editor, to bring together some of the many loose strings concerned with the role of trace elements in relation to nutrition and cancer.

This book, which is unusual rather than comprehensive, is refreshing because most of its contributors are enthusiasts. If it was their purpose to convince the reader that metals and inorganic materials are of great importance as carcinogens, as cancer chemotherapeutic agents and as nutrients that prevent cancer, one has to say that they failed. Nevertheless, their combined efforts make interesting reading.

F. J. C. ROE

[1] Bridbord, K., Decoufle, P., Fraumeni, J. F. & 6 others. Estimates of the Fraction of Cancer in the United States Related to Occupational Factors. Manuscript dated 15 September 1978; circulated by National Institute for Occupational Safety and Health, Washington, D.C.